# Advanced trauma life support course for medical students. A new era?

**DOI:** 10.3389/fsurg.2022.1025920

**Published:** 2022-12-26

**Authors:** Panteleimon Vassiliu, Andreas Mavrogenis, Christos Theos, Panagiotis Koulouvaris, Ioannis Massalis, Angelos Geranios, Christos Bartsokas, Michael Gerazounis, Konstantinos Tepetes, Apostolos Kamparoudis, Spyros Stergiopoulos, Michael Stavropoulos, John Androulakis

**Affiliations:** ^1^ 4th Surgical Clinic, Attikon University Hospital, Athens, Greece; ^2^ 1st Orthopedic Clinic, Attikon University Hospital, Athens, Greece; ^3^ Orthopedic Clinic, Metropolitan Hospital, Pireas, Greece; ^4^ Department of Surgery, Nafplio General Hospital, Nafplion, Greece; ^5^ General Surgery & Critical Care Consultant, Khobash General Hospital, Najran district, Kingdom of Saudi Arabia; ^6^ Department of Thoracic Surgery, General State Hospital of Nikaia “Saint Panteleimon”, Nikea, Greece; ^7^ Department of Surgery, University General Hospital of Larissa, Larissa, Greece; ^8^ Surgical Clinic, Ippokrateio - General Hospital of Thessaloniki, Thessaloniki, Greece; ^9^ 1st Propaedeutic Surgical Clinic, Ippokrateio - General Hospital of Athens, Athens, Greece; ^10^ Department of Surgery, General University Hospital of Patras, Rio, Greece

**Keywords:** medical education, ATLS (Advanced trauma life support), medical students, trauma, short term course

## Abstract

**Introduction:**

Trauma represents a major public health issue and is one of the leading causes of death and disability worldwide. A systematic approach toward dealing with trauma patients was facilitated through the ATLS program, which has become a milestone in trauma care. Our new ATLS course for medical students was set in motion in 2015. Our aim was to make medical students familiar with trauma patients interactively, through a program like ATLS, and here we present the results of this endeavor.

**Methods:**

A two-day ATLS-Medical Student (MS) course was offered from November 2015 to July 2018, and analysis was performed retrospectively on the data gathered over a three-month period through online questionnaires. Before graduating, 261 newly qualified medical doctors were interviewed and evaluated as part of the ATLS course.

**Results:**

After the course, the vast majority of medical students (251 MSs; 96.16%) felt more capable of managing severely injured patients and 58% of students felt that the medical services they offered were better due to the ATLS training. Regarding the educational fee for the course, 56.7% of the students reported that they felt the fee of 100 euros was fair.

**Discussion:**

The interactive format of the course, which differs from more traditional methods of teaching, has been endorsed by medical students. Though they lack clinical experience, that does not prohibit them from acquiring more specialized or specific knowledge, enabling them to excel. Most of the students improved their skillset either in theoretical knowledge, practical skills, or even in the emotional component of the course, i.e., dealing with treating a severely injured patient. It was decided that the program would be re-evaluated and extended to all Greek Medical Schools.

**Conclusion:**

The advantage of providing doctors with trauma training at the beginning of their careers is evident. For that reason, it was decided that the program would be re-evaluated and extended to all Greek Medical Schools.

## Introduction

Trauma is one of the most important public health problems worldwide, with enormous personal, psychological, social, and economic consequences (1). Injuries affect all areas of society, regardless of age, gender, race, or economic status, and they can have severe outcomes in terms of the loss of productive years of life, temporary or permanent disability, and financial cost (2). Data suggest that a systematic and scientifically documented solution to the problem is needed. Despite the proven improvement in the treatment of seriously injured patients in recent decades, based on international medical practices, trauma remains one of the main causes of death and disability (3).

The management of severe trauma evolves during conflicts (4), for example, during the apartheid era in Soweto, where an organized civilian hospital accepted thousands of wounded patients during a brief period (5, 6). The ATLS course was first introduced in the 1980s (7) and has since been providing doctors worldwide with the basic knowledge and skillset required for initial trauma management, relieving the global trauma burden.

With the support of the Stavros Niarchos Foundation (SNF), an international humanitarian organization that decided to support medical students (MS) rather than financially independent physicians (8), we attempted to be innovative within the ATLS context. After we obtained permission from the American College of Surgeons, we delivered the full ATLS course to MSs (ATLS-MS) immediately before their graduation. Even though medical school students lack clinical experience (9), the course could benefit their overall performance as young resident or rural-service doctors. In our study, we aim to examine the absorption of this complex new knowledge by the MSs.

## Methods

From November 2015 to July 2018, we offered an ATLS-MS to Athens and Patras Universities' Medical Schools. The interested students were accepted based on the following criteria:

• Being in the last year of their medical studies*
• Having passed all their subjects in the previous years
  * Students retained the privilege of being able to apply to the course up until one day before their graduation.
  * They had to complete the course within a maximum of one year of their graduation day.

The schedule of ATLS courses during the study period consisted of one course in 2015, nine in 2016, ten in 2017, and five in 2018, with an outcome of 392 MSs being trained in total. Of those, 266 (67.85%) responded to the online survey after three reminders. Out of them, five were excluded as they never completed the course with a successful final exam. This left us with 261 MSs for further analysis. A comparable number (350 in total) of students were trained in the Patras center, with only 78 of them completing the survey, and only a small fraction, five out of 78, completing the report without significant missing values. They were therefore excluded from the analysis. This may be attributed to the fact that there were technical issues with the server at the Patras center and subsequent delays in gathering the data. We therefore decided to provide preliminary results as a single-center analysis.

The MSs were obliged to pay a minimal educational fee to show their genuine interest, evaluate their motivation, realize their obligation to prepare for the course, and proceed with their application and commitment to the course. The applicants were provided with all the ATLS study material (9th edition) and, after a month, they undertook a formal ATLS course, MCQ exam, and evaluation from the instructors. The potential outcomes were, as in the ATLS course, either “Instructor potential” for those excelling in performance, “pass”, or “fail” in theory, practice, or both. Students gave their written feedback, evaluating each lecture, skill, or simulation session.

‘Funds from the supporting organization were originally offered for a one-year pilot evaluation period to test the project. With judicious financial management, the pilot period was extended to 2.5 years, with no additional funding.

After each course, the report was given to the parent organization (local and metropolitan ATLS). The MSs’ contact details and the records of their efforts at ATLS were kept – with their approval – for future reference.

In 2019, an online survey was sent to all participants *via* email. The survey was resent to the non-responders, with two additional reminders over a period of three months. The questionnaire referred to demographics (age; gender; year and city in which they took the course; their clinical or non-clinical orientation; and their motivation to take the ATLS) and their evaluation of the course quality and its impact on their medical practice (capability to manage severely injured patients; what they liked most; time devoted to preparing for the course; their theoretical, technical, and emotional maturity before and after the course; whether and how the ATLS helped them; whether the fee was fair; how the course compared with other courses they had attended during their medical studies; their opinion regarding the faculty; and whether they would recommend the course to peers).

The responses were analyzed using statistical package, SPSS v.25. The statistically significant level was kept at 0.05. Categorical variables were compared with the chi-square test and continuous variables with the *t*-test.

## Results

For the present analysis, the results yielded pertain only to the Athenian ATLS center.

Based on the questionnaire, the average age of the 261 participants of the Athenian center was 25.4 years, the majority being women (63.22%), and 54.4% of participants attended the course in order to be prepared for mandatory rural service (Table 1).

**Table 1:** Students’ motivation to participate in the course.

Motivation	Number of MS	%
Preparing for mandatory rural service^a^	142	54,4
Preparing for a career abroad	59	22,6
Recommended by friends	23	8,8
Value for money	16	6,1
Preparedness to act in times of need	13	4,9
Other (CME certificate, curiousness)	8	2.9
Total	261	100%

^a^ Every Greek physician after completion of medical school must carry out a year's service in remote, rural areas as a general practitioner.

After the course, the majority (251 MSs; 96.16%) felt more capable of managing severely injured patients. The skill stations and the scenarios were the sections they liked the most in their training (136 MSs; 52.11%), followed by the “emotional retraining” in the simulation stations (76 MSs, 29.12%), then the theory and lectures (39 MSs, 14.94%) in the 9th ATLS edition. Ten MSs (3.83%) achieved the best performance in the management of the severely wounded.

On average, they studied for 19 days before the course.

Their theoretical, technical (skill), and emotional maturity regarding trauma management improved; this was measured on a scale of 1 point (minimum) to 5 points (maximum) before and after the course (Table 2).

**Table 2:** Course evaluation by medical students.

	Before ATLS (no. of MS)	After ATLS (no. of MS)	*P-value*
Theory Knowledge (grading)			
1 (less)	35	0	
2	96	24	
3	68	77	<0,001
4	58	97	
5 (more)	4	63	
Average grade ± SD	2.61 ± 0.9	3.76 ± 1.1	0.097
Skill/Technical Knowledge (grading)			
1 (less)	110	3	
2	89	82	
3	47	74	<0.001
4	12	76	
5 (more)	3	26	
Average grade ± SD	1.89 ± 0.9	3.15 ± 1.0	<0.001
Emotional maturity (grading)			
1 (less)	127	6	
2	78	63	
3	40	80	<0.001
4	13	82	
5 (more)	3	30	
Average grade ± SD	1.8 ± 0.9	3,25 ± 1.0	<0.001

Regarding the effects of ATLS on their practice, 58% of the MSs felt that their medical services, not only trauma-related, were better due to ATLS training, while 25.67% sensed that ATLS training solely improved their medical knowledge in trauma. A fifth (19.16%) of the MSs stated that the course helped them in practice to save a life. One MS (0.38%) stated that it was not helpful. None of the participants selected the answer “The seminar was a waste of time” (Table 3).

**Table 3:** Daily practice function in relation to their previous medical practice (clinical or not) and motivation for taking the course.

		Daily practice function *N* (%)		*P-value*
		*n = 193*	*n = 68*	
		Improved	Not improved	
Experience	Clinical	160 (82.9)	41 (60.3)	0.001
	Non-clinical	5 (2.6)	3 (4.4)	
	Any	28 (14.5)	24 (35.3)	
Motivation	Rural Service	114 (59.1)	28 (41.2)	0.047
	Work abroad	41 (21.2)	18 (26.5)	
	Good recommendation	13 (6.7)	10 (14.7)	
	Other motivation	25 (13.0)	12 (17.6)	

Regarding the educational fee, 56.7% of the MSs reported that the fee of 100 euros was fair, 14.94% stated that it should have been free of charge, 16.86% stated that it was invaluable and should be taken at any cost, and the remainder stated that a price above the current educational fee would have been appropriate.

Compared with other courses MSs have taken in the past, 29.89% of MSs stated that ATLS was the best, and 62.07% reported that it was one of the best seminars they had ever attended. Two MSs stated that they had no similar experience with which to compare it. The course was average for 6.5% of the students and poor for two students (0.77%).

According to 47.13% of the MSs, the faculty played a crucial role in their educational experience; 27.59% of the MSs were inspired by the faculty's professionalism and focus on human values, 24.9% of the MSs found the role of the faculty was neutral, and one MS (0.38%) stated that the faculty had a negative effect on the educational outcome of the course.

The course should be taken by all physicians according to 51.72% of the present cohort. It should be offered to all MSs, as stated by 28.35% of the MSs, and to young doctors specifically, as stated by an additional 18.77% of the MSs. Two students stated that it should be offered to experienced doctors, and one said that it was a waste of time and should not be recommended.

## Discussion

The initial interest of MSs in the course evolved through “word of mouth” dissemination and gradually saw a significant increase; no budget was devoted to advertisement other than an online platform to facilitate the application process. The interest of the MS community rose to the extent that MSs from all the Greek Medical Schools (seven universities in total) were traveling at their own expense to one of the two cities where the course was offered (Patras and Athens). There was no other reason to exclude data from the Patras course except the technical issues mentioned above, which led to a lot of missing values that could eventually skew our results.

During their studies, MSs are exposed to multiple complicated subjects related to human physiology and pathology. Severe trauma management teaching in ATLS combines many of those curricular elements into an integrated and clinically applicable entity. Its major advantage is that it is completely oriented around medical practice and retrains the physician psychologically to confront emotionally disturbing clinical entities with confidence.

An initial effort to teach ATLS to MSs was made by the University of Manitoba, in Winnipeg, in 1992 (10), when the course was delivered to 4th-year medical students for the first time and was considered for incorporation into the medical curriculum. The number of MSs taking the course was much less (90) in the Winnipeg study, but the results were comparable to those of our study; the success rate was comparable in the MS and mature doctor (MD) ATLS courses, most of the participants suggesting the course should be provided earlier in the medical career (senior MS or junior MD), and the most appreciated sessions of the course being the practical sessions (skill stations, scenarios, simulation). In our study, the Instructor Potential (IP) rate in the MS group was not statistically different from the IP rate in the MD group. Candidates who did not successfully complete the course in the MS group were, statistically, significantly more than those who failed in the MD group (9,6% vs. 3,2%). Nevertheless, the fact that MSs became aware of trauma management, a subject that is not included in the medical school curriculum, and more than 90% of them became ATLS certified, is a substantial improvement to their medical skillset, as it incorporates the skill and emotional retraining of ATLS (11). This carries the potential for better medical services to be offered to the community. In addition, in a country without a trauma system, trauma awareness among medical doctors potentially intensifies the demand to develop an organized system and the need for personnel to run it.

The MSs faced no significant difficulties in incorporating theoretical knowledge in the interactive manner offered at ATLS, and in addition, appreciated the practical part more, in which they were exposed to realistic clinical scenarios simulating what they could face in real-life practice. The emotional retraining on how they should react, backed up by the acquired theory, led them not only to pass the course but to appreciate it in their early medical life, as they managed severe disease with a confidence that they could not have imagined at the end of their formal medical studies (12, 13).

In Table 3, it is evident that students who selected the clinical path experienced a remarkable improvement in their daily practice functions compared to those who did not have a post or those who had a non-clinical one. In addition, those who followed formal rural service also benefitted more compared to those planning to move abroad or those who attended just because of it had been recommended to them.

Moreover, there was a statistically significant difference in the pre- and post-course student self-evaluation score in “emotional handling” of a trauma patient (pre 1.75 ± 0.9 vs. post 3.25 ± 1.0; *p-value *< 0.001) and also in technical skills (pre 1.9 ± 0.9 vs. post 3.15 ± 1.0; *p-value *< 0.001), showing a great improvement following the course. In contrast, for theoretical knowledge, a statistically significant difference between scores before and after the course was not observed (pre 2.61 ± 1.0 vs. post 2.79 ± 1.1; *p-value *= 0.097).

It seems that the theory of trauma management in theoretically well-trained medical students is easily acquired with a few weeks of dedicated studying. What is invaluable for them is the skill station and the simulated scenarios involving severely injured patients, which offers them the chance to develop their technical skill and increases their ability to transform overwhelming emotional stress – when faced with a dying patient and the responsibility to act as an expert – into an effective, potentially life-saving action. The same idea is applied in the 10th edition of ATLS, where the students come to the educational center for the skill and simulation sessions after completing the theoretical study and theory exams at home. The idea of training future clinicians in a more “clinical” way, incorporating scenarios and skill and simulation stations could be applied in the medical curriculum, based on the aforementioned results.

Emotional maturity is thought to capture a characteristic of individuals that can help to evaluate how they handle their own emotions and interact with others in both personal and professional relationships, especially in stressful settings. Doctors who had increased emotional maturity had higher patient satisfaction rates, along with better stress management and reduced burnout (11, 14, 15).

The most significant point of the proposed idea to teach the ATLS course to MSs is the preparation of young doctors for treating trauma effectively early in their careers. This will assist in the development of a nationally organized trauma system, and, above all, positively impact the outcome and epidemics of trauma in the country. Our study was not designed to evaluate all those effects, so this is a task to accomplish in the future.

There are specific strengths associated with the current study. Firstly, data were selected using a random sample of the population of medical students. Secondly, the outcome was clearly stated. However, there are also certain limitations. This is an observational study and, therefore, we cannot establish causal relationships between the independent factors examined in this study. In addition, data from another important ATLS center were missing, making our sample smaller.

## Conclusion

The ATLS course was accepted with enthusiasm by final-year medical students. MDs who are ATLS-trained at the beginning of their careers will be the leading force in the development of a nationally organized trauma system and will assist in the incorporation of this standardized course into the medical curriculum.

## Data Availability

The raw data supporting the conclusions of this article will be made available by the authors, without undue reservation.

## In Memoriam of

Michael Gerazounis, dedicated ATLS Director.

## References

[1] World Health Organization. Guidelines for trauma quality improvement programmes. Geneva: World Health Organization, Department of Violence and Injury Prevention and Disability (2009).

[2] American College of Surgeons. Committee on Trauma. Advanced trauma life support for doctors – student course manual. 8th ed Chicago: American College of Surgeons (2008).

[3] Committee on Trauma. American College of Surgeons. Resources for optimal care of the injured patient 2014. Chicago: American College of Surgeons (2014).

[4] Kauvar DS Holcomb JB Norris GC Hess Jr. Fresh whole blood transfusion: a controversial military practice. J Trauma. (2006) 61(1):181–4. 10.1097/01.ta.0000222671.84335.64 16832268

[5] Degiannis E Levy R Velmahos GC Mokoena T Daponte A Saadia R. Gunshot injuries of the liver: the baragwanath experience. Surgery. (1995) 117(4):359–64. 10.1016/S0039-6060(05)80053-4 7716715

[6] Demetriades D Charalambides D Lakhoo M Pantanowitz D. Gunshot wound of the abdomen: role of selective conservative management. BJS. (1991) 78(2):220–2. 10.1002/bjs.1800780230 2015480

[7] Advanced Trauma Life Support. American College of Surgeons. Committee of Trauma. https://www.facs.org/quality-programs/trauma/atls.

[8] Stavros Niarchos Foundation. https://www.snf.org/en/grants/grantees/a/atls-hellas/program-support/.

[9] Saultz J. Clinical experience in medical school. Fam Med. (2018) 50(1):5–6. 10.22454/FamMed.2018.601141 29346693

[10] Ali J Howard M. The advanced trauma life support course for senior medical students. Can J Surg. (1992) 35(5):541–5. PMID: 1393872

[11] Wagner PJ Moseley GC Grant MM Gore JR Owens C. Physicians’ emotional intelligence and patient satisfaction. Fam Med. (2002) 34(10):750–4. PMID: 12448645

[12] Wise H McMillian L Carpenter C Mohanty K Abdul W Hughes A. Implementation of novel trauma and life-support course for undergraduate medical students. Orthopaedic proceedings 2022 mar (vol. 104, No. SUPP_3, pp. 1-1). The British Editorial Society of Bone & Joint Surgery.

[13] Mastoridis S Shanmugarajah K Kneebone R. Undergraduate education in trauma training: the students’ verdict on current teaching. Med Teach. (2011) 33(7):585–7. 10.3109/0142159X.2011.576716 21696289

[14] Cofer KD Hollis RH Goss L Morris MS Porterfield JR Chu DI. Burnout is associated with emotional intelligence but not traditional job performance measurements in surgical residents. J Surg Educ. (2018) 75:1171–9. 10.1016/j.jsurg.2018.01.021 29483035

[15] Shahid R Stirling J Adams W. Promoting wellness and stress management in residents through emotional intelligence training. Adv Med Educ Pract. (2018) 9:681–6. 10.2147/AMEP.S175299 30310341 PMC6165721

